# Topics of Public Interest

**Published:** 1914-06

**Authors:** 


### TOPICS OF PUBLIC INTEREST

**Cost of Pasteurizing Milk.** The U. S. Dept. of Agriculture estimates 3.13 mills per gallon, and about twice as much for cream. The "holder" process, 30 minutes at 135-145 is cheaper and more reliable than the "flash" process, 165 for a moment.

---

**Lack of Room for Feeble Minded Children.** The State Charities Aid Assn. (Homer Folks, Sec., 105 E. 22, N. Y.) reports the N. Y. City institution on Randall's Island as having 400 more inmates than the buildings were planned to accommodate; Rome and Craig Colonies overcrowded and with long waiting lists; Letchworth Village not completed. It is estimated that there are 32,000 feeble minded in the state, of whom 4900 are in institutions designed for their care, 5000 in reformatories and prisons. It is hoped that the legislature will appropriate $300,000 to provide approximately 1000 beds, to relieve the immediate congestion. At the Rome State Custodial Asylum, the per capita cost is $2.39 per week. The cost in boys' reformatories is $4.66; in girls' $5.47. Adequate provision for this defective class is not only a philanthropy which the state should undertake; it is an economy, both in the sense that the feeble minded can be cared for more cheaply on a large scale and that by proper direction they can be made more or less self-supporting, and in the sense that institutional control prevents crime and limits the tendency to propagation of a defective class. However, in view of the fact that taxation has already reached the point of being a heavy burden, care should be taken not to relieve individual families of responsibility in this direction, unless the circumstances justify such action.

---

**Veto of Christian Science Bill.** On April 24, Gov. Glynn vetoed the bill permitting Christian Scientists and other practitioners who do not use drugs to practice without medical examination. He stated that he believed that Christian Science healers should be allowed to practice among followers of this faith but that the bill was so broad in its terms as to open the gates to all sorts of medical pretenders. We certainly agree with Gov. Glynn that this bill should have been vetoed, and for the reasons stated. Drugs have played the main part in the practice of the healing art until recently; they are still an important item; we doubt very much whether essentially chemic disturbances of health can ever be successfully treated except by chemic means, in other words, by drugs. But, the abstention from the use of drugs in the practice of the healing art, instead of demanding lower requirements, increases the standard of technical information and skill which the practitioner should possess. In any line of human activity, standards must be established and questions of fact and opinion settled by the best representatives of the majority engaged in that line. Drugless healers, pipeless plumbers, insurance companies that do not insure, automobilists that do not observe the majority opinion and the law regarding traffic, must all expect to be "persecuted."

Subscribers will please not stop their subscription and the board of censors are requested to be lenient but we are going to express the heterodox opinion that Gov. Glynn may be right in his contention that a bill allowing healers to practice among Christian Scientists, would be justified. We would except minors from such a provision, also persons of unsound mind or non compos mentis as the result of disease or injury, unless they had previously signified their willingness to abide by the results. But we are inclined to believe that the question of fact at issue between the medical profession and various other conservative persons, and the Christian Scientists, can be settled only by actual experience, that it is ultimately more humane to allow suffering and death from neglect of medical science, now, and on a scale sufficiently large to be instructive, than to force the reluctant to yield to the majority opinion by legislation and thus to give the opposing side the highly valuable logical argument of martyrdom. The medical profession has been openly charged with mercenary motives in its endeavors to protect the public health by requiring high standards of scientific efficiency and by excluding eccentric cults of single ideas from the management of physical ailments. This charge is absurd. If the intent of the profession were to force the laity into employing its services, the attempt at compulsion would defeat its own end and while treatment by charlatans and one-idea men reduces, immediately, the number of persons who would otherwise seek regular medical services, in the long run it increases the need for such services. We are almost ready to follow the slogan: "Hic populus vult decipi; decipiatur in nomine Diaboli." This people wants to be taken in; in the Devil's name, let it be.

---

**Pure Food Law.** The Godfrey bill, signed by the Governor, makes dealers, restaurant and hotel keepers, equally responsible with manufacturers for the sale or serving of adulterated or misbranded food, false statement of manufacturer on a package being included as misbranding. At first thought, this might seem unjust but allowance would undoubtedly be made by any court, in the case of an innocent dealer, to whom goods have been misrepresented. Usually, price, circumstances of sale, standing of manufacturers, etc., would prevent the deception of any wholesale purchaser who was not willing to be deceived. A plan of insurance against suits and fines might even be put into operation.

---

**Expenses of Medical Society of the State of New York.** Former President Campbell recently stated the principal expenses to be approximately as follows: legal defense, $4,900; journal, $4,400; directory, $6,000.

---

**Steuben County Tuberculosis Hospital.** A committee has been appointed to select a site and prepare plans. Dr. E. E. Webster of Woodhull represents the medical profession.

---

**Niagara County Tuberculosis Hospital.** It is proposed to use the present county hospital near Lockport for tuberculosis patients, as soon as the new almshouse is finished. At present, 31 tuberculous patients are being cared for outside the county. There is some opposition to the plan proposed and, in turn, the supervisors are opposed to outside interference.

---

**Nurses' Home and Maternity Hospital,** have been recommended by the State Board of Charities for the Lockport City Hospital.

---

**Free Antitoxin** has been discontinued in Ashtabula, owing to the abuse of the charity by persons in good circumstances.

---

**Reports of Death Due to Anti-Variolar and Anti-Typhoid Vaccination Incorrect.** Press reports have stated that two Niagara Falls teachers died as the result of anti-variolar vaccination. The fact is that only one teacher died in that city during the past winter and her death was due to chronic nephritis—authority, Dr. Linsly R. Williams, Deputy Commissioner of Health. On page 648 of the May issue, we noted the death of a national guardsman, who was said to have died of typhoid, three weeks after a prophylactic inoculation. Aside from an error in name, the press notices should have stated that he died three months after inoculation and that the cause of death was malignant endocarditis and general sepsis. This case having been disposed of, we believe that there has been no case in American military surgical experience in which death from typhoid has occurred after inoculation. Foreign experience has, however, shown that inoculation is not an absolute preventive although the mortality from typhoid is much less than in the uninoculated.

**Thermic Treatment for Gonorrhoea.** On page 431, February issue, we noted the claim of priority by Dr. J. A. Fulton of Astoria. Lieut. Col. Charles E. Woodruff, of New Rochelle, writes that he used hot water irrigation in 1901, published the method in the Medical Record, and later found that Hoff of the U. S. Army had used the same method 20 years earlier.

---

**Diphthongs.** Some of our readers have asked why we cling to diphthongs instead of helping the cause of simplified spelling. Dr. Achilles Rose, in a recent letter, says in very courteous language and in approving spirit what may be interpreted to mean that this journal is the only one left that is a crank on the subject of medical nomenclature. Incidentally, he gives two illustrations of the reason for clinging to the original diphthongs: Anaesthesia means nudity and anaemia windiness. Paediatrics, clearly means the treatment of children. Pediatrics might be construed to mean a treatment by fetters or bandages, or by steering a case, or, with due allowance to the tendency to mix Latin and Greek, as a treatment of the feet and there is no question but that many persons do think that the term refers to the management of various forms of talipes and has been extended to include other deformities of growth and the management of children's diseases in general. But the real reason for preferring diphthongs is this: English is a very new and very mixed language. With due allowance for gradual development from its origins and for the relatively rapid and considerable changes that all modern languages have had forced on them by the necessity for new words to keep up with discoveries and inventions and by the tendency to mutual influence in the way of idioms, it may almost be said that English was constructed between 1350 and 1550. At any rate, English of the former date is almost unintelligible and that of the latter date merely quaint. Aside from the purely artificial languages, volapuk, esperanto, etc., there has never been such a rapid and extensive linguistic development anywhere in the world. English is spelled according to the original phonetic schemes of its several components and pronounced according to no single scheme. The only way in which it can be written phonetically, under present conditions, is by a complicated plan of silent, indicator letters and this plan is only approximately followed in actual practice. It has 12 clearly recognized vowel sounds and 5 letters to represent them. It has 22 clearly recognized consonant sounds. For five of these, arbitrary combinations of letters are required, one indeed, having no characteristic method of representation at all. There are only 10 consonant letters that can be reasonably assumed, if encountered in an unfamiliar word, to have a definite sound. Under these circumstances, we feel that the attempt to patch up the English language and put it on a phonetic basis is hopeless. The only feasible method is the radical one of building up a new alphabet of at least 34 letters and cutting loose entirely from traditions, as is done in some systems of short hand. Under these conditions, we feel that spelling should be a matter of authority and of history, especially in the sense of showing the derivation of a word.

**Safety First.** Buffalo is placarded with Safety First signs and is having the usual number of automobile accidents of various kinds, the streets being still used as playgrounds. One trouble about fly swatting, cleaning-up weeks, safety first and various other campaigns is that they are so largely literary and oratoric. We endeavor to avoid poetry but the following hits the nail on the head:

The shades of night were falling fast
As through a crowded street, there passed
A truck of eighty odd horsepower
Which bore at thirty miles an hour
A banner labeled "SAFETY FIRST."

**A Physician the Victim of Smallpox.** Dr. W. J. Pirsh, of Fredonia is quarantined on account of a mild attack, of unknown origin.

**Typhoid in Dunkirk.** 26 cases were reported for March, 45 for April. Chlorid of lime has been employed since the early part of May, in treating the water.

**Centenary of Birth of Dr. Edward Mott Moore.** This will be celebrated in Rochester, July 15. The Park Board has contributed $2,000 and the city is expected to erect a statue. This is a fitting honor to a great man, all the more significant of appreciation because of the lapse of time since his death. We had the pleasure and benefit of assisting Dr. Moore in 1888, as interne, were impressed with the success of methods antedating aseptic surgery and still more so with the progressive spirit which led this man, in his old age, after careful consideration, to adopt antiseptic methods.

**State Board Statistics.** The Journal of the A. M. A., May 23, gives its usual, exhaustive annual report. 6,435 physicians were examined in 1913, including 794 graduated in 1908 or earlier. Of recent graduates, 56.4% were examined in the state in which they graduated, with 11.9% of failures. 18.6% failed in states other than the one of graduation. The general average of failures was 18.6%, 28.1% for graduates of 1908 and earlier, 16.5% for graduates between 1909 and 1913, 10.2% for graduates of 1913. Quantity sometimes implies quality; 12.6% failed of colleges having 100 or more examined; 37.5% of colleges having between 50 and 100 examined; 17.1% of still smaller colleges. There are 99 colleges in the United States now giving medical degrees, but only 65 recognized by 80% or more of individual state boards. 19 colleges are recognized by less than half the state boards. It is safe to say that in the near future, we shall have to deal with not over 80 colleges. Four states require two years' of collegiate work of graduates of the present year; three one year; 11 others will require one or two years, of such preliminary study applying to graduates in 1915 to 1919. New York is not in either of these lists and the example, broadly speaking, is set by the west for the east to follow. The whole report should be carefully studied.

---

**Too Literal.** The pessimistic articles on medical economics have brought forth a circular offering a job at $15 per week.

---

**Too Literal.** Health Commissioner Fronczak, of Buffalo, is objecting to mitigation of quarantine rules by prominent physicians for prominent families. He ought to realize that certain noble principles of this land of equality were intended for the Fourth of July, the same as certain principles of religion and morality were intended for Sunday. The every-day application of such principles causes trouble.

---

**Physician Convicted of Manslaughter.** Dr. Martin E. Griffith of Monessen, Pa., has been convicted on account of the death of Wm. J. Robinson a music teacher (not the editor of the Critic and Guide), following cudaheyzation.

---

**Vivisection Reports.** Dr. Walter B. Cannon, Chairman of the Bureau of the A. M. A. for the Protection of Medical Research, urges writers to make definite statements regarding anaesthesia, to prevent distortion of reports by antivivisectionists who, in the absence of such statements will consider the experience as cruel.

In this connection, we may allude to the trial of Dr. Joshua E. Sweet, of the University of Pennsylvania, who with five others, was indicted on a charge brought by the Women's Society for the Prevention of Cruelty to Animals. In one sense, this charge was an entirely proper one, since it waived the entire matter of experiment in the direct way, and related to the care of animals, pending and following experiment. The case resulted in a disagreement and, so far as one can judge from the testimony, it was prejudiced, and without reasonable basis. But, granting that the charge was made in good faith, the general contention that all animals kept in captivity whether for the production of meat, wool, milk, etc., or for exhibition purposes, or as domestic pets, or in the interests of science and for therapeutic products, should be regularly fed and watered and otherwise humanely dealt with, is proper and reasonable.

---

**Biologic Products Illustrated by Moving Pictures.** The H. K. Mulford Company will have at the A. M. A. meeting at Atlantic City, an interesting exhibit of methods of preparing and using antitoxins, bacterins, vaccines, etc.

---

**Harrison Anti-Narcotic Bill.** Senator Knute Nelson offered an amendment which practically prohibited physicians, dentists and veterinarians from dispensing narcotic drugs and which prevented a physician from sending such a drug by messenger or ordering a nurse to administer it in his absence. Senator Nelson states that this amendment was offered at the request of C. H. Huhn, of Minneapolis, Secretary of the Minnesota Retail Druggists' Association. If true, this represents a little too close regard for the pharmacist's interests.

---

**Ratio of Physicians.** The new Polk Directory will contain, as did the issue of 1912, the names of upward of 140,000 physicians. This is for the U. S., including possessions and Canada, representing a population of about 108 million in 1910 and about 115 million at present. The general ratio is, therefore, about 1 physician to 820 population. For average states, the ratio is, of course, much lower but, with allowance for the number of retired and non-participating physicians included, is approximately 1 to 700.

---

### Mortality Statistics of the United States.

(Bureau of the Census.)

**Death Rate From Principle Causes.** Tuberculosis markedly decreased, although it still causes a vast number of unnecessary deaths—90,360, or 149.5 per 100,000 in 1912, (10.8) of the total mortality. Next came organic diseases of the heart, with 86,179 deaths (adding endocarditis, they slightly exceeded tuberculosis), acute nephritis and Bright's disease (62,267), pneumonia (51,495), congenital debility and malformations (48,596), cerebral hemorrhage and softening (46,797), cancer (46,531), and diarrhea and enteritis of infants under 2 years of age (42,482). There were 63,385 deaths from external causes, of which 49,755 were due to accident, 9,656 to suicide, and 3,954 to homicide. The suicide rate (16 per 100,000 population) was slightly lower than that for 1911 (16.2) and is the same as the average for 1906 to 1910.

Typhoid fever, with 9,987 deaths (16.5 per 1,000), showed a notable decrease from the preceding year (12,451) and a most gratifying reduction from the average rate for the five-year period 1901-1905 (32). In other words, it has been cut in half in the last decade, although our rate is still high as compared with some European countries.

